# Identification of Inhibitory Activities of Dietary Flavonoids against URAT1, a Renal Urate Re-Absorber: In Vitro Screening and Fractional Approach Focused on Rooibos Leaves

**DOI:** 10.3390/nu14030575

**Published:** 2022-01-28

**Authors:** Yu Toyoda, Tappei Takada, Hiroki Saito, Hiroshi Hirata, Ami Ota-Kontani, Youichi Tsuchiya, Hiroshi Suzuki

**Affiliations:** 1 Department of Pharmacy, The University of Tokyo Hospital, 7-3-1 Hongo, Bunkyo-ku, Tokyo 113-8655, Japan; ytoyoda-tky@umin.ac.jp (Y.T.); hiroki.saito@sapporoholdings.co.jp (H.S.); suzukihi-tky@umin.ac.jp (H.S.); 2 Frontier Laboratories for Value Creation, Sapporo Holdings Ltd., 10 Okatome, Yaizu, Shizuoka 425-0013, Japan; xjtpt341@gmail.com (H.H.); ami.ota@sapporoholdings.co.jp (A.O.-K.); yoichi.tsuchiya@sapporoholdings.co.jp (Y.T.)

**Keywords:** SLC22A12, quercetin, fisetin, uricosuric activity, anti-hyperuricemia, functional food, transporter, uric acid, health promotion, rooibos tea

## Abstract

Hyperuricemia, a lifestyle-related disease characterized by elevated serum urate levels, is the main risk factor for gout; therefore, the serum urate-lowering effects of human diets or dietary ingredients have attracted widespread interest. As Urate transporter 1 (URAT1) governs most urate reabsorption from primary urine into blood, URAT1 inhibition helps decrease serum urate levels by increasing the net renal urate excretion. In this study, we used a cell-based urate transport assay to investigate the URAT1-inhibitory effects of 162 extracts of plant materials consumed by humans. Among these, we focused on *Aspalathus linearis*, the source of rooibos tea, to explore its active ingredients. Using liquid–liquid extraction with subsequent column chromatography, as well as spectrometric analyses for chemical characterization, we identified quercetin as a URAT1 inhibitor. We also investigated the URAT1-inhibitory activities of 23 dietary ingredients including nine flavanols, two flavanonols, two flavones, two isoflavonoids, eight chalcones, and a coumarin. Among the tested authentic chemicals, fisetin and quercetin showed the strongest and second-strongest URAT1-inhibitory activities, with IC_50_ values of 7.5 and 12.6 μM, respectively. Although these effects of phytochemicals should be investigated further in human studies, our findings may provide new clues for using nutraceuticals to promote health.

## 1. Introduction

Hyperuricemia is a lifestyle-related disease with an increasing global prevalence [1]. Sustained elevation of serum urate is a major risk factor for developing gout [2], the most common form of inflammatory arthritis. Therefore, serum urate management within appropriate ranges is important for health care. In the human body, uric acid is the end-product of purine metabolism because functional uricase (the urate-degrading enzyme) is genetically lost [3]. Consequently, serum urate levels are determined by the balance between the production and excretion of the urate—the predominant form of uric acid under physiological pH conditions. The kidney is responsible for the daily elimination of approximately two-thirds of urate [4]. However, the net proportion of urate secreted into the urine is only 3–10% of the urate filtered by the renal glomerulus [5]. This is because most of the filtered urate is re-absorbed from primary urine into the blood by renal proximal tubular cells through the urate transporter 1 (URAT1)-mediated pathway [6]. Therefore, inhibition of this route increases the net urinary excretion of urate, resulting in decreased serum urate.

URAT1, also known as SLC22A12, is a physiologically important renal urate re-absorber; its dysfunction causes renal hypouricemia type 1 [6,7], a genetic disorder characterized by impaired renal urate reabsorption, associated with extremely low serum urate levels (serum urate ≤ 2 mg/dL [8,9]; normal range: 3.0 to 7.0 mg/dL). Among the already identified urate reabsorption transporters that are expressed on the renal cell apical membrane, URAT1 has the highest influence on serum urate levels. Accordingly, in hyperuricemia, this urate transporter is considered a pharmacological target of some anti-hyperuricemic agents that promote renal urate excretion. The uricosuric effect based on URAT1 inhibition forms the mechanism of action for SUA-lowering drugs such as benzbromarone [6] and lesinurad [10]. Based on this information, daily consumption of food ingredients with URAT1-inhibitory activity may bring a beneficial effect on serum urate management in individuals with high serum urate levels. Hence, the exploration of URAT1-inhibitory ingredients in the human diet has received increasing attention. Previously, we and other groups have successfully identified food ingredients from *Citrus* flavonoids [11], coumarins [12], wood pigments [13], and fatty acids [14]. As just described, natural products are promising sources of URAT1-inhibitory compounds, encouraging us to explore such ingredients in various ordinary plants purchased in the market.

We herein investigated the URAT1-inhibitory activities of 162 dietary plant products employing a mammalian cell-based urate transport assay. Via screening plant extracts followed by liquid–liquid extraction and column chromatography, we successfully identified quercetin, a flavonol, as a novel URAT1 inhibitor with a half-maximal inhibitory concentration (IC_50_) of 12.6 µM from rooibos (*Aspalathus linearis*) leaves. Focusing on other dietary flavonoids, we further investigated their effects on URAT1 function, and among the tested compounds in this study, we identified fisetin as the strongest URAT1 inhibitory ingredient with an IC_50_ of 7.5 μM. The experimental procedures described below and the information obtained on URAT1-inhibitory activities in various plant extracts will be useful for further identification of natural product-derived URAT1 inhibitors.

## 2. Materials and Methods

### 2.1. Materials and Resources

The critical materials and resources are summarized in Table 1. All other chemicals were of analytical grade and were commercially available. All authentic chemicals were re-dissolved in dimethyl sulfoxide (DMSO) (Nacalai Tesque, Kyoto, Japan). Each inhibition assay was carried out with the same lot of the expression vector for URAT1 (URAT1 wild-type inserted in pEGFP-C1) or mock (empty vector, i.e., pEGFP-C1), derived from our previous study [14]. Urate transport assay (see below) using these vectors was used and validated in previous studies [11,14,15]. The plant materials (Table A1) were purchased, between July 2016 and July 2017, from local supermarkets in Shizuoka, Japan.

### 2.2. Preparation of Plant Ethanolic Extracts

Plant extracts were prepared as described in our previous studies [16,17], with some modifications. In brief, after fruits were cleaned, the peels and pulps were separated carefully. The fresh and dried materials (see Table A1) were chopped finely using a knife and ground using a mill (Crush Millser IFM-C20Gb) (Iwatani, Tokyo, Japan), respectively. In the next extraction step, the preprocessed plant material (approximately 50 g) was immersed in 100 mL of ethanol, sonicated for 5 min, and stirred for 30 min at room temperature, and the suspension was passed through a filter paper. Then, the filtrate was evaporated and dissolved in methanol. The extract was dried, weighed, dissolved in DMSO at 2 mg/mL (2000 ppm), and stored at −20 °C until use. Next, 5 μL of the resulting solution was mixed with 245 μL of Cl^−^-free transport buffer (Buffer T2: 125 mM Na-gluconate, 25 mM HEPES, 5.6 mM D-glucose, 4.8 mM K-gluconate, 1.3 mM Ca-gluconate, 1.2 mM MgSO_4_, 1.2 mM KH_2_PO_4_, and pH 7.4), and this clear liquid (250 μL) was used for the urate transport assay (final concentration: 20 ppm with 1% DMSO) as described below.

### 2.3. Cell Culture

Human embryonic kidney 293-derived 293A cells were maintained in DMEM—Dulbecco’s Modified Eagle’s Medium (Nacalai Tesque) supplemented with 10% fetal bovine serum (Biowest, Nuaillé, France), 2 mM L-Glutamine (Nacalai Tesque), 1 × Non-Essential Amino Acid (Life Technologies, Carlsbad, CA, USA), and 1% penicillin–streptomycin (Nacalai Tesque), at 37 °C in a humidified atmosphere of 5% (*v*/*v*) CO_2_ in air.

As described previously [14], the plasmids for URAT1 expression or mock were transfected into 293A cells using polyethylenimine “MAX” (PEI-MAX) (Polysciences, Warrington, PA, USA). In brief, 293A cells were seeded onto twelve-well cell culture plates at a concentration of 0.92 × 10^5^ cells/cm^2^. After 24 h, each vector was transiently transfected into the cells (1 μg of plasmid/5 μL of PEI-MAX/well). At 24 h after transfection, the culture medium was replaced with fresh one.

### 2.4. Urate Transport Assay Using URAT1-Expressing 293A Cells

The urate transport assay using transiently URAT1-expressing 293A cells was conducted as described in our previous studies [11,14,18], with minor modifications. Briefly, 48 h after plasmid transfection, cells were washed twice with Buffer T2 and then pre-incubated in Buffer T2 for 15 min at 37 °C. Then, the buffer was exchanged with pre-warmed fresh Buffer T2 containing radiolabeled urate ([8-^14^C]-uric acid; final concentration, 5 μM) with or without the test compound at the indicated concentrations (0, 0.3, 1, 3, 10, 30, 100, 300, or 500 μM); the cells were further incubated for 20 s at 37 °C; as vehicle control, 1% DMSO was used in this study. Subsequently, the cells were washed five times with ice-cold Buffer T2; then, the cells were lysed with 0.2 M NaOH solution (500 μL/well) on ice. The resulting lysates were neutralized with 1 M HCl solution (100 μL/well). The radioactivity in the lysate was then measured using a liquid scintillator (Tri-Carb 3110TR) (PerkinElmer, Waltham, MA, USA) for DPM (disintegrations per minute) counting. Using a Pierce BCA Protein Assay Kit (Thermo Fisher Scientific, Kanagawa, Japan), protein concentration was determined. Urate transport activity was calculated as the incorporated clearance (μL/mg protein/min): (incorporated level of urate [DPM/mg protein/min]/urate level in the incubation mixture [DPM/μL]). URAT1-dependent urate transport activity was calculated by subtracting the urate transport activity of mock (control) cells from that of URAT1-expressing cells.

Urate uptake was examined in the presence of several concentrations of each test compound to determine their IC_50_ values. Then, URAT1-mediated transport activities were expressed as a percentage of the control (100%). Based on the calculated values, fitting curves were obtained according to the following formula using the least-squares method in Excel 2019 (Microsoft, Redmond, WA, USA):

(1) Predicted value %=100−Emax×CnEC50n+Cn

where E_max_ is the maximum effect; EC_50_ is the half-maximal effective concentration; C is the concentration of the tested compound; n is the sigmoid-fit factor. Lastly, based on these results, the IC_50_ value was calculated as previously described [11,14].

### 2.5. Fractionation of Rooibos Tea Leaves Extract

To purify the active ingredients for URAT1-inhibitory activity in the ethanolic extract of rooibos tea leaves, liquid–liquid extraction and column chromatography were conducted according to previous studies [16,17], with some modifications as described below.

First, the dried crude ethanolic extract of rooibos tea leaves was subjected to sequential liquid–liquid extraction using a solvent series with increasing polarity (*n*-hexane, ethyl acetate, and *n*-butanol). In brief, the ethanolic extract was mixed in approximately 500 mL of distilled water and added to a glass separatory funnel. Subsequently, an equal volume of *n*-hexane was added to the solution and mixed well for partitioning. After formation of the dual-phase, the *n*-hexane phase was collected; the remaining water phase was then shaken with the same volume of ethyl acetate. After the ethyl acetate phase was collected in a similar manner, the water phase was further partitioned with *n*-butanol. Finally, the *n*-butanol phase and bottom layer (aqueous phase residue) were collected separately. After evaporation process, the phases were reconstituted in an appropriate solvent prior to use in the urate transport assay for evaluation of URAT1-inhibitory activities and/or further separation by medium-pressure liquid chromatography (MPLC) as follows.

The ethyl acetate fraction, which was reconstituted in hexane and ethyl acetate for normal-phase chromatographic purification, was separated into 14 subfractions (Fr.#1–Fr.#14) by MPLC using a dual-channel automated flash chromatography system (EPCLC-W-Prep 2XY) (Yamazen, Osaka, Japan) on a disposable Silica-gel packed column with high throughput purification (Universal column premium Silicagel L, 40 g) (Yamazen, Osaka, Japan). Separation was conducted in the linear gradient elution mode with solvent A (hexane), solvent B (ethyl acetate), and solvent C (methanol) [solvent A:solvent B:solvent C (*v/v*): 0–4 min 90:10:0, 4–8 min 90:10:0 to 60:40:0, 8–12 min 60:40:0, 12–32 min 60:40:0 to 0:100:0, 32–35.8 min 0:100:0, 35.8–36 min 0:0:100, 36–37 min 0:100:0, 37–53 min 0:100:0 to 0:50:50, 53–60 min 0:50:50] at a flow rate of 20 mL/min, with UV monitoring at 280 nm using an equipped UV detector. All subfractions obtained were evaporated to dryness and stored at −20 °C. Before use, they were reconstituted in DMSO (2 mg/mL).

### 2.6. Chemical Characterization

For qualitative determination of the isolated compounds, chromatographic separation and subsequent mass spectrometry (MS) (or MS/MS) analyses were performed using an LC-quadrupole time-of-flight (Q-TOF)-MS/MS system comprising an HPLC instrument [Agilent 1200 Series equipped with a diode array and multiple wavelength detector (DAD) (G1316A)] coupled with an Agilent 6510 Q-TOF (Agilent Technologies, Santa Clara, CA, USA) as described previously [16,17], with minor modifications. In brief, separation was performed on a Zorbax Eclipse Plus C18 column (1.8 μm, 2.1 mm × 100 mm; Agilent Technologies) maintained at 40 °C under gradient mobile conditions with a mixture of solvent C (0.1% formic acid in water) and solvent D (acetonitrile) (solvent C:solvent D (*v*/*v*): 0–8 min 95:5 to 5:95, 8–12 min 5:95) at a flow rate of 0.5 mL/min. The detection range of DAD was set from 190 to 400 nm; the MS detection system was operated in the positive ionization mode at an MS scan range of *m/z* 100–1100. Ionization was performed using a heated electrospray ionization probe with the following source parameters: gas temperature, 350 °C; drying gas, 12 L/min; nebulizer, 55 psi; Vcap, 3.5 kV. Peak analysis was conducted using the Agilent MassHunter Workstation software (version B.03.01; Agilent Technologies).

### 2.7. Statistical Analysis

We performed all statistical analyses using Excel 2019 with Statcel4 add-in software (OMS Publishing, Saitama, Japan). Different statistical tests were used for different experiments, as described in the figure legends, which include the number of biological replicates (*n*). In brief, when analyzing multiple groups, the similarity of variance between groups was compared using Bartlett’s test. When passing the test for homogeneity of variance, a parametric Tukey–Kramer multiple-comparison test for all pairwise comparisons or a Dunnett’s test for comparisons with a control group was used; otherwise, a non-parametric Steel test was used for comparisons with a control group. Likewise, to examine the concentration-dependent decrease in the URAT1 activity in the presence of extracts, a parametric Williams’s multiple-comparison test or a non-parametric Shirley–Williams’s multiple-comparison test was used. To investigate the inhibitory effect of each authentic chemical on URAT1 function (vs. the vehicle control indicated as 100%) in the screening stage, a one-sample *t*-test (two-sided) was employed. Statistical significance in this study was defined as *p* < 0.05 or 0.01.

Each experiment was designed to use the samples required to obtain informative results and sufficient material for subsequent procedures. All experiments were monitored in a non-blinded manner. No specific statistical test was employed to pre-determine the sample sizes which were empirically determined in the present study.

## 3. Results

### 3.1. Screening the URAT1-Inhibitory Activities of Plant Extracts

**Figure 1:**
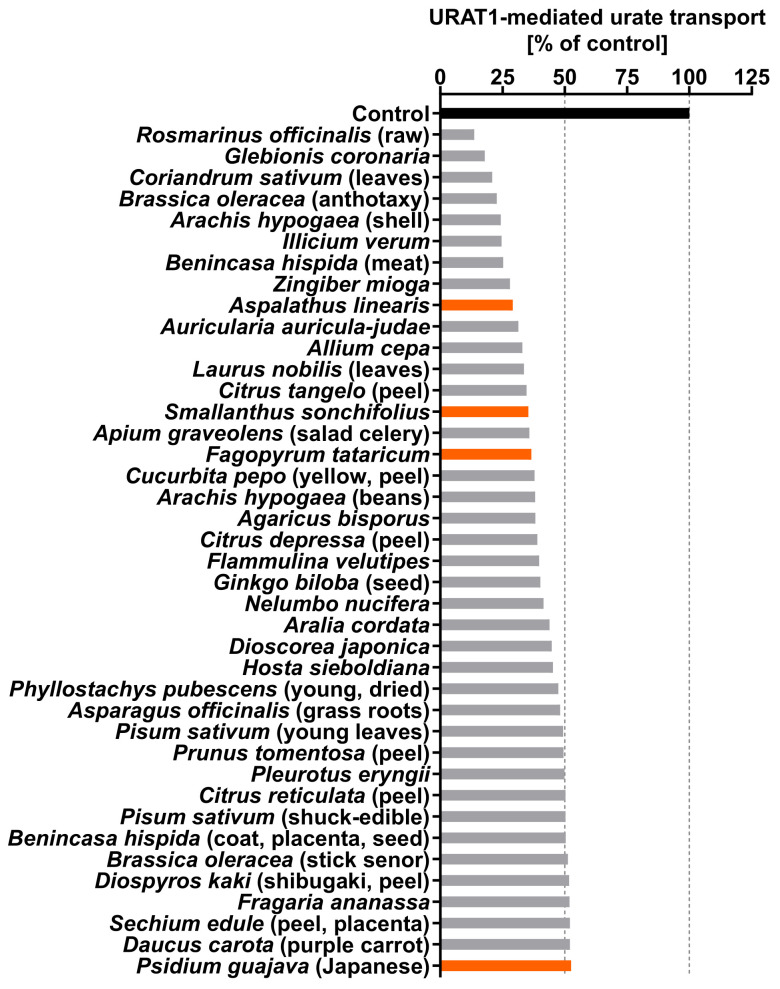
Screening of the inhibitory effects of various plant extracts on URAT1 function. The effects of each ethanolic extract (20 ppm), which was dried and finally dissolved in dimethyl sulfoxide (DMSO) at 2000 ppm (see Section 2.2.), on the URAT1-mediated [^14^C]-urate transport was investigated using the cell-based urate transport assay; as the vehicle control, 1% DMSO was used. *Orange* indicates herbal tea sources. All data are expressed as % of the vehicle control (*n* = 1, each sample). This figure shows the results of the top 40 samples of the tested extracts (total 162); all data are listed in Table A2.

For the URAT1-inhibitory properties of natural products, we herein focused on various plants in the human diet including vegetables, fruits, and tea leaves. Each plant sample (Table A1) was extracted with ethanol, and a total of 162 ethanolic extracts were subjected to in vitro screening for URAT1-inhibitory activity at 20 ppm (Table A2). The top 40 samples (approximately 25%) of the tested extracts (Figure 1) included four kinds of herbal tea sources: rooibos tea (*Aspalathus linearis*), yacon tea (*Smallanthus sonchifolius*), Tartary buckwheat tea (*Fagopyrum tataricum*), and guava leaf tea (*Psidium guajava*). As the rooibos leaf extract was the most active among these, and because rooibos tea is globally consumed [19], we next explored the ingredients responsible for URAT1-inhibitory activity in rooibos tea leaves.

### 3.2. Fractionation and Isolation of the Aspalathus linearis (Rooibos Leaves) Extract

**Figure 2:**
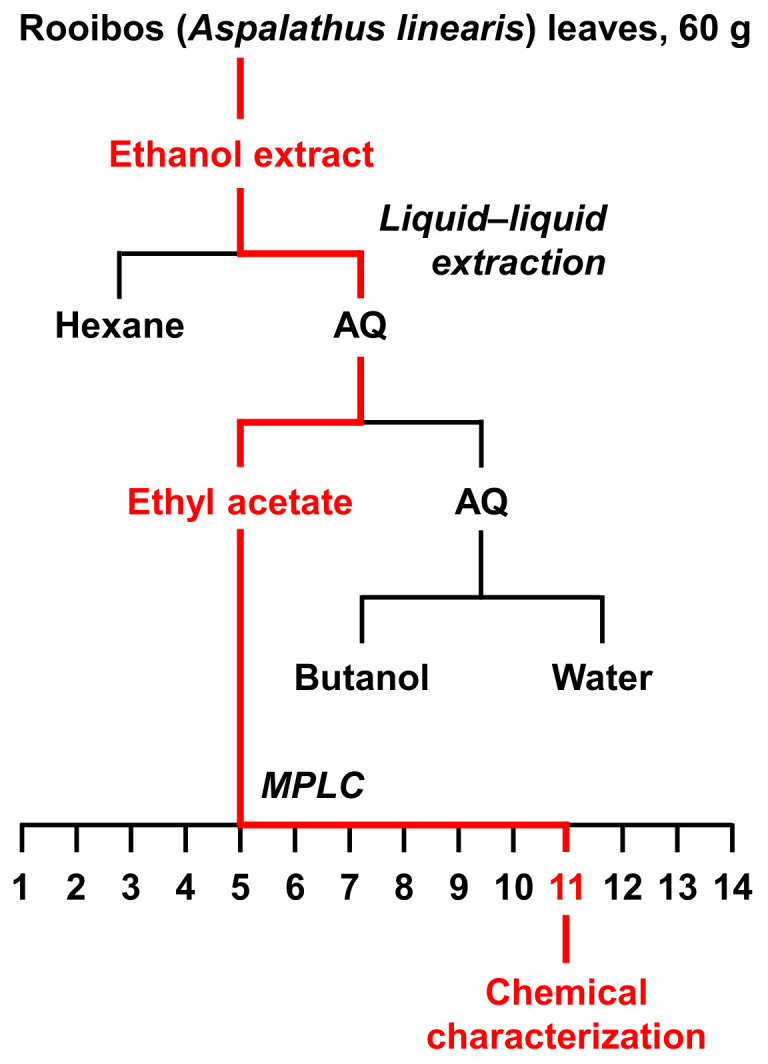
A flow chart of extraction and isolation for rooibos (*Aspalathus linearis*) leaves. In each separation procedure, the fraction with the highest URAT1-inhibitory activity is colored in red. AQ, aqueous layer; MPLC, medium pressure liquid chromatography.

To determine the URAT1-inhibitory ingredients in the ethanolic extract of rooibos leaves, further fractionation was carried out using liquid–liquid extraction and subsequent column chromatography (Figure 2). For this purpose, 60 g of rooibos leaves were newly extracted using ethanol.

**Figure 3:**
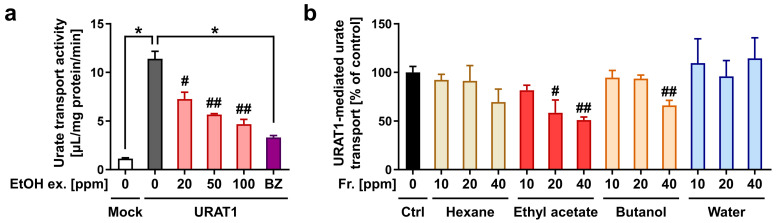
URAT1-inhibitory activity of the ethanolic extraction of rooibos leaves and each fraction obtained by liquid–liquid extraction; 1% dimethyl sulfoxide was used as the vehicle control. (**a**) Concentration-dependent URAT1 inhibitory activity of the ethanolic extraction (EtOH ex.); 0 ppm means only vehicle treatment. Mock, empty vector-transfected cells for the detection of background activity for urate transport; BZ, benzbromarone (final concentration 2.5 μM), a well-known URAT1 inhibitor, was used as the positive control. All data are expressed as the mean ± S.E.M., *n* = 4. #, *p* < 0.05; ##, *p* < 0.01 with concentration-dependent decreasing tendency vs. the control (Shirley–Williams’s multiple-comparison test); *, *p* < 0.05 between the indicated groups (Steel test) (**b**) URAT1 inhibitory activity of each fraction (Fr.). All data are expressed as % of the vehicle control (Ctrl) and the mean ± S.E.M., *n* = 3–4. #, *p* < 0.05; ##, *p* < 0.01 with a concentration-dependent decreasing tendency vs. the control (Williams’ test in each fraction category).

**Figure 4:**
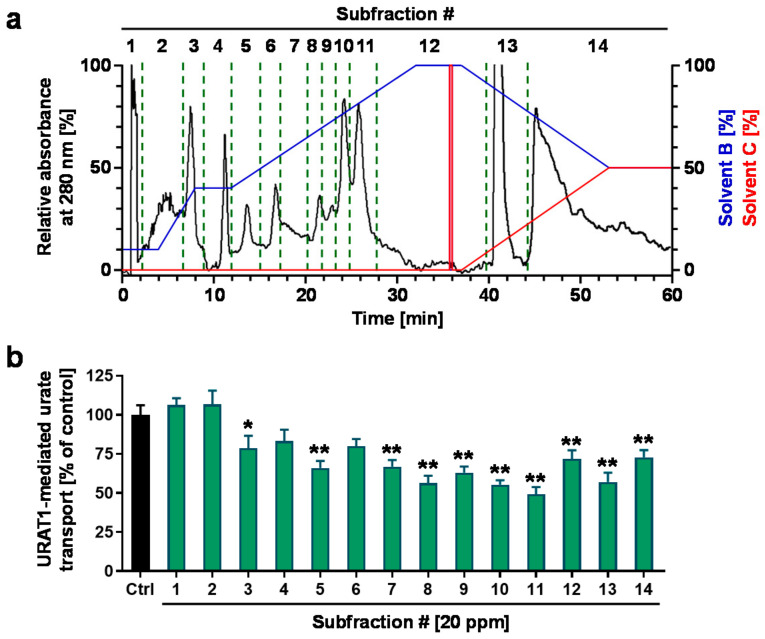
URAT1-inhibitory activity of each subfraction from the ethyl acetate fraction of the ethanolic extract of rooibos leaves. (**a**) A preparative MPLC chromatogram for separating the ethyl acetate fraction. The chromatogram was recorded at 280 nm. Blue and red lines indicate the linear gradients of solvent B (ethyl acetate) and solvent C (methanol), respectively. (**b**) URAT1-inhibitory activity profile of each subfraction (20 ppm) obtained from the column chromatography; 1% dimethyl sulfoxide was used for the vehicle control. All data are expressed as % of the vehicle control and the mean ± S.E.M.; *n* = 9 (Ctrl, control), 5 (the others). #, fraction number; *, *p* < 0.05; **, *p* < 0.01 vs. control (Dunnett’s test).

Prior to fractionation, we confirmed the concentration-dependent URAT1-inhibitory effect of the ethanolic extract (Figure 3a). The extract was then separated sequentially into *n*-hexane, ethyl acetate, *n*-butanol, and water-soluble fractions. Among the four fractions, the ethyl acetate fraction had the highest URAT1-inhibitory activity (Figure 3b). Both the *n*-hexane and water fractions showed little inhibitory activity, whereas the ethyl acetate fraction exhibited URAT1-inhibitory activity in a concentration-dependent manner. Additionally, the *n*-butanol fraction inhibited URAT1-mediated urate transport only at the maximum concentration employed in this study (40 ppm); however, its effect was weaker than that of the ethyl acetate fraction. Therefore, we further separated the ethyl acetate fraction by column chromatography to obtain a total of 14 subfractions (Fr.#1–#14) based on the monitored absorbance chromatogram (Figure 4a), as described in the *Materials and Methods* section (Section 2.5). The URAT1-inhibitory activity of each subfraction was then analyzed; among the 14 subfractions, Fr.#11 showed the highest URAT1-inhibitory activity (Figure 4b). Therefore, we focused on this subfraction for further analyses.

### 3.3. Structural Characterization of the Putative URAT1 Inhibitor Derived from Rooibos Leaves

**Figure 5:**
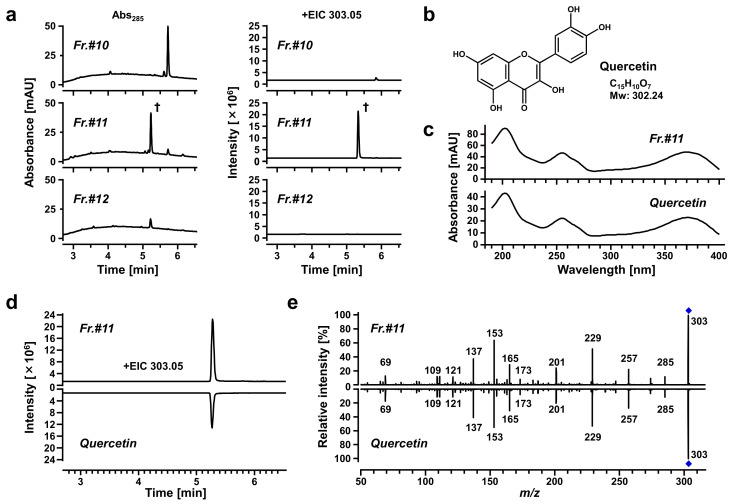
Chemical characterization of a URAT1-inhibitory activity-guided fraction from the ethanolic extract of rooibos leaves. Each subfraction and authentic quercetin (lower panels in (**c**–**e**)) were analyzed using a high-performance liquid chromatography instrument coupled with a diode array and multiple wavelength detector (LC-DAD), and a quadrupole time-of-flight-mass spectrometry system (LC-Q-TOF-MS). (**a**) Purity verification of the isolated ingredient in Subfraction #11 (Fr.#11) by spectrometric analyses. *Left panels*, UV chromatograms recorded at 285 nm. *Right panels*, LC-Q-TOF-MS extracted ion chromatograms (EICs; at *m/z* 303.0506 in the positive ESI spectrum). †, a specific peak in Fr.#11 with a retention time of 5.298 min. (**b**) Chemical structure of quercetin. (**c**–**e**) Comparison of obtained data between Fr.#11 and quercetin; (**c**) DAD spectrum; (**d**) EIC; (**e**) information regarding the fragment ions derived from MS/MS analyses.

We used spectrometric analyses to determine the purity of the target subfraction (Fr.#11) and to obtain structural information about the candidate active ingredients (Figure 5). The results of LC-DAD analyses supported that the ingredient yielding the main peak in the chromatogram of Fr.#11 was almost completely isolated from the other subfractions (Figure 5a, left); subsequent LC-Q-TOF-MS analyses revealed that based on the obtained accurate mass information (*m/z* 303.0506 in the positive ion mode with a retention time of 5.298 min), the elemental composition of the target analyte was determined as C_15_H_10_O_7_ (Δ−2.14 ppm from [M+H]^+^) (Figure 5a, right). Based on the polarity of ethyl acetate, the sub-fractionation source (ethyl acetate fraction) was considered to contain flavonoids characterized by a 15-carbon skeleton (C_6_-C_3_-C_6_). Moreover, the compositional formula (C_15_H_10_O_7_) was consistent with that of quercetin, and a previous study has identified quercetin in rooibos leaves [20]. Based on this information, we hypothesized that the main component of Fr.#11 could be quercetin (Figure 5b). To test this hypothesis, we conducted spectroscopic analyses and found that Fr.#11 and authentic quercetin were identical in their photoabsorption spectrum (Figure 5c), retention time, the accurate mass of the parent ion (Figure 5d), and MS/MS spectrum (Figure 5e). Thus, the isolated substance should be quercetin.

### 3.4. Identification of Quercetin as an Active Ingredient with URAT1-Inhibitory Activity

**Figure 6:**
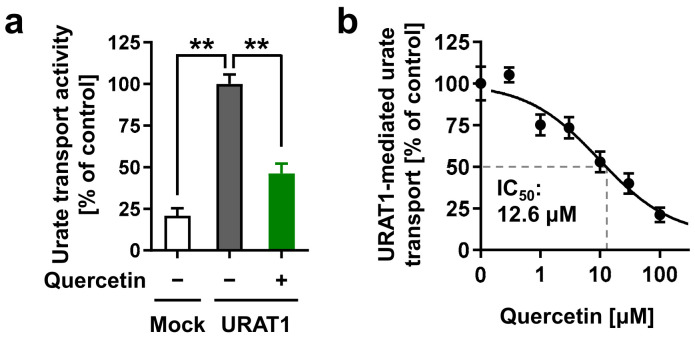
Effects of quercetin on the URAT1 function. (**a**) Inhibitory effects of quercetin (300 μM) on URAT1-mediated urate transport. (**b**) Concentration-dependent inhibition. All data are expressed as % of the vehicle control (1% dimethyl sulfoxide) and the mean ± S.E.M.; *n* = 4. **, *p* < 0.05 (Tukey–Kramer multiple-comparison test).

To determine whether quercetin could be responsible for inhibiting URAT1 function, we investigated the effect of quercetin on the urate transport activity of URAT1 (Figure 6). As expected, at the experimentally maximum concentration (300 μM), quercetin inhibited URAT1 (Figure 6a). Further investigation of its concentration-dependent inhibitory effects revealed an IC_50_ of 12.6 μM (Figure 6b). Based on these results and the determined structural characteristics (Figure 5), we concluded that quercetin was an active ingredient in Fr.#11.

### 3.5. URAT1-Inhibitory Activities of Various Dietary Flavonoids

**Figure 7:**
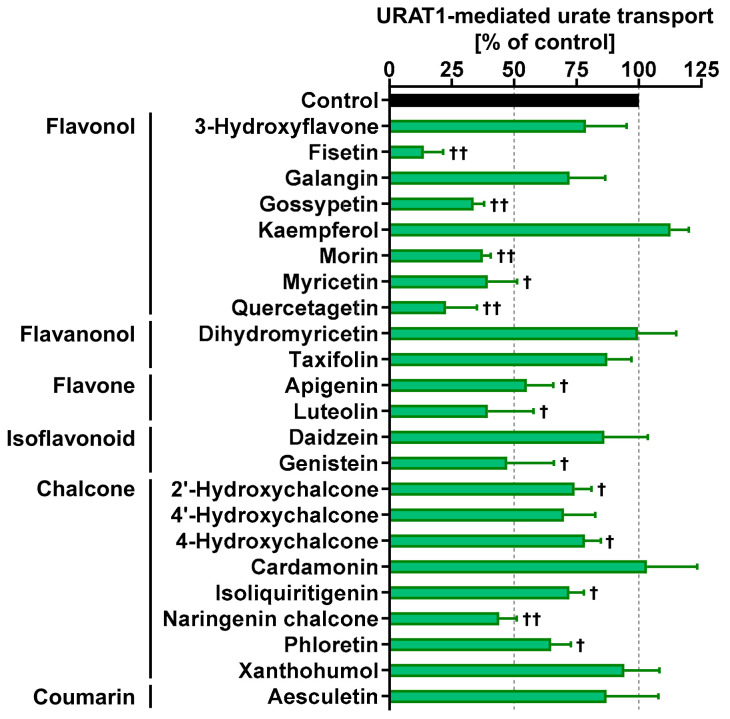
URAT1-inhibitory activities of each food ingredient at 100 μM; 1% dimethyl sulfoxide was used as the vehicle control. All data are expressed as % of the vehicle control and the mean ± S.D.; *n* = 3. †, *p* < 0.05; ††, *p* < 0.01 vs. vehicle control (two-sided one-sample *t*-test).

**Figure A1:**
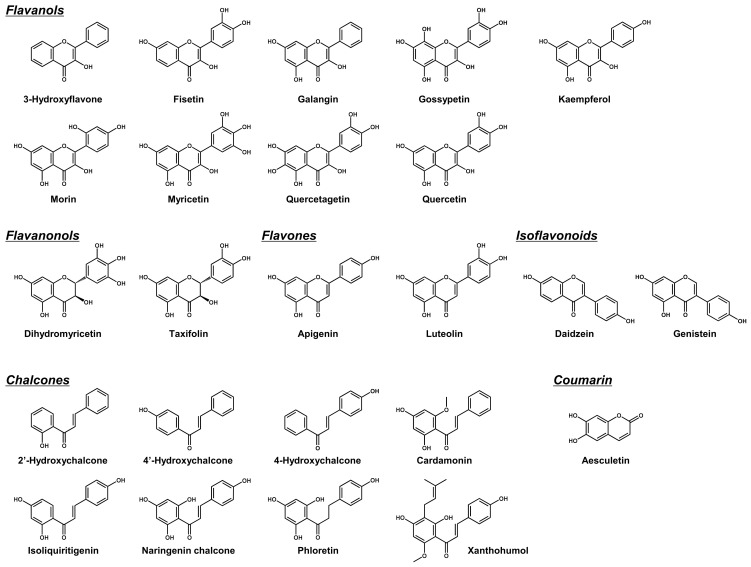
Chemical structures of authentic chemicals tested in this study.

Finally, we investigated whether other dietary flavonoids of interest, including nine flavanols, two flavanonols, two flavones, two isoflavonoids, and eight chalcones, have URAT1-inhibitory activities (Figure 7). Additionally, we also tested aesculetin, a coumarin identified in rooibos leaves [20]. The chemical structures of the selected natural compounds are summarized in Figure A1. At 100 μM, eight of the tested authentic chemicals (fisetin, gossypetin, morin, myricetin, quercetagetin, luteolin, genistein, and naringenin chalcone) lowered the URAT1-mediated urate transport to less than 50% of that in the control group. Our results were qualitatively consistent with a previous report showing that morin is a URAT1 inhibitor [13]. Moreover, based on our previous study, the URAT1-mediated urate transport activity in the presence of 100 μM naringenin chalcone (44.0 ± 7.1% of that of the control) (Figure 7) was higher than that in the presence of 100 μM naringenin (17.9 ± 7.7%) [11], suggesting that naringenin chalcone has a weaker URAT1-inhibitory activity than naringenin, a metabolite of naringenin chalcone. Thus, we focused on the other six flavonoids in our subsequent analyses.

**Figure 8:**
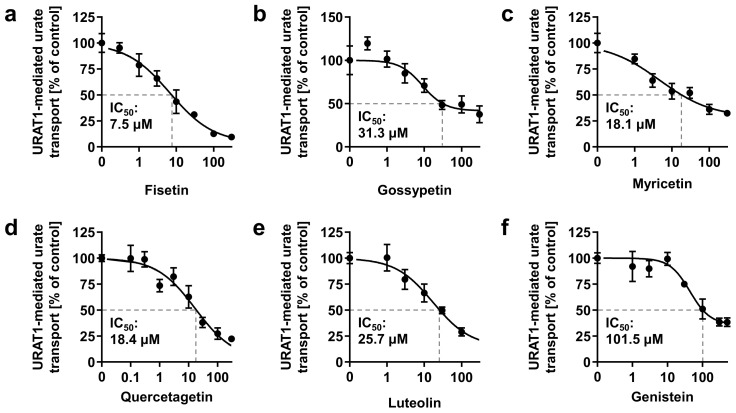
Concentration-dependent inhibition of URAT1-mediated urate transport by (**a**) fisetin; (**b**) gossypetin; (**c**) myricetin; (**d**) quercetagetin; (**e**) luteolin; (**f**) genistein. The x-axis indicates drug concentrations (μM). All data are expressed as % of the vehicle control and the mean ± S.E.M.; *n* = 4 (**a**–**e**), 3 (**f**).

Further investigation of the concentration-dependent inhibitory effects of the six flavonoids on URAT1 determined their IC_50_ values (Figure 8). Genistein exhibited the highest IC_50_ value among the tested samples, and its value was consistent with the results of flavonoid screening (Figure 7). Based on these IC_50_ values and that of quercetin, fisetin was the strongest URAT1 inhibitor among the seven dietary flavonoids examined (Figure 8a), whereas quercetin was second to fisetin (Figure 6b). Based on the structural difference between fisetin and quercetin (Figure A1), the presence of a hydroxyl group at the 5-position of the flavanol skeleton could somewhat negatively affect the URAT1-inhibitory effect. Interestingly, a contrasting effect was observed in the case of the isoflavone skeleton, as shown for daidzein and genistein (Figure 7). Although further studies are needed to clarify the quantitative structure–activity relationship, our findings provide a better understanding of small molecule-dependent URAT1 inhibition.

## 4. Discussion

In this study, we screened the inhibitory effects of the ethanolic extracts of various dietary plant materials on the function of URAT1 as a urate transporter (Figure 1). Among the plants, we focused on rooibos leaves and identified quercetin as an active ingredient responsible for the URAT1-inhibitory activity in the extract (Figure 2, Figure 3, Figure 4, Figure 5 and Figure 6). Moreover, to extend our understanding of the interaction between URAT1 and flavonoids, 24 dietary flavonoids were further investigated (Figure 7 and Figure 8). Although some previous studies have examined the effect of certain flavonoids with respect to their effect on URAT1-mediated urate transport [11,13], to the best of our knowledge, this is the first study to comprehensively address the inhibitory effect of dietary flavonoids on URAT1 function.

Flavonoids are well-known ingredients of natural products and have received considerable attention for their health-promoting and/or potential therapeutic properties in many diseases based on a broad spectrum of biological functions including anti-oxidative, anti-inflammatory, neuroprotective, and anti-cancer activities [21,22]. Although the present study was limited to in vitro evaluations, our findings regarding the potential uricosuric activities of flavonoids may extend the possibilities of their nutraceutical application. In particular, fisetin and quercetin, which exhibited the smallest and second-smallest IC_50_ values against urate transport by URAT1, respectively, are relatively well studied and are some of the most prevalent plant flavonoids [23,24,25]. However, to our knowledge, few studies have investigated their effects on the renal handling of endogenous substances such as urate. Further studies are thus required to deepen our understanding of this issue. Further, fisetin and quercetin are abundantly found in fruits and vegetables such as apples and onions [23]; fisetin is also abundant in strawberries and teas [24]. Hence, the effects of dietary habits including such plant-based foods on serum urate levels and renal urate handling are of significant interest.

Although little information is available on the effects of quercetin on urate handling in humans, a human study (randomized, double-blinded, placebo-controlled, cross-over four-week intervention trial) demonstrated that daily supplementation with 500 mg quercetin as a single tablet, which contained the bioavailable amount of quercetin as present in approximately 100 g of red onions, significantly reduced (mean difference, −0.45 mg/dL) the plasma uric acid concentrations (mean, 5.5 mg/dL) in healthy males [26]. In contrast, the previous study confirmed the urinary excretion of quercetin, but did not sufficiently investigate the effect of quercetin on renal urate handling—no parameters on renal urate clearance or fractional excretion of uric acid were reported; only the urinary uric acid output over a 24-h period (24 h-UUA) was documented. Given that no significant difference in the amount of 24 h-UUA was found between before and after the quercetin treatment, despite the serum urate-lowering effect, renal urate clearance might have been influenced by quercetin. Based on the inhibitory activity of quercetin against xanthine oxidoreductase (XOD, an essential enzyme of uric acid formation) [27], the serum urate-lowering effect was considered to have been mainly associated with suppressed uric acid production; however, quercetin might also have enhanced renal urate excretion. Although such a dual inhibitory activity will be welcome, it is necessary to understand the activity that majorly contributes to the serum urate-lowering effect in the context of combination with serum urate-lowering drugs (urate synthesis inhibitors or uricosuric agents), for a more effective application.

Our findings also highlight the potential health benefits of rooibos-based food products. Today, especially for health-conscious people, the global popularity of rooibos tea seems to depend on its caffeine-free status, comparatively low tannin content, and anti-oxidative activity as potential health-promoting properties [28]. The influence of continuous consumption of rooibos tea on the serum urate levels and the risk of urate-related diseases remains to be elucidated; however, in addition to our findings demonstrating URAT1-inhibitory activity in rooibos extract (Figure 1) and rooibos flavonoids (Figure 6 and Figure 7), a previous study reported that aspalathin, a C-glycosyl dihydrochalcone contained in rooibos, inhibits XOD [29]. Given this information, additional human studies and/or epidemiological studies will be helpful to address the serum urate-lowering potential of rooibos extracts.

Some limitations of this study and possible future directions are described below. First, although we successfully identified quercetin as an active compound for URAT1-inhibitory activity in rooibos extract, other ingredients may have contributed to the activity based on the results of the bioactivity-guided fractionation approach (Figure 3 and Figure 4). Some of these might overlap with the dietary flavonoids tested in this study. Second, the other plant extracts that we could not handle further in this study may be good sources for exploring additional compounds for URAT1-inhibition. Third, to extrapolate our findings to humans, bioavailability and in vivo levels in nutraceutical-achievable situations as well as the effects of metabolic conversion on the URAT1-inhibitory activities of dietary flavonoids should be examined. Despite the need for further studies, as most previous studies were conducted to find plant-derived bioactive compounds with potential anti-hyperuricemia activity in the context of XOD inhibition [30], our study focusing on their potential uricosuric activity will facilitate progress in nutrition research, contributing to the treatment and prevention of hyperuricemia.

## 5. Conclusions

We found that the ethanolic extract of rooibos leaves inhibited the urate transport activity of URAT1. From this plant extract, we successfully identified quercetin, a natural compound considered safe for humans, as an active ingredient. Moreover, we expanded our understanding of the inhibitory effects of dietary flavonoids and chalcones on URAT1 function in a comprehensive manner. These effects of phytochemicals need further investigation in human studies; however, our findings may provide new clues for promoting health through appropriate serum urate maintenance.

## 6. Patents

Yu Toyoda, Tappei Takada, Hiroki Saito, Hiroshi Hirata, Ami Ota-Kontani, and Hiroshi Suzuki have a patent pending related to the work reported in this article.

## Figures and Tables

**Table 1:** Key resources.

REAGENT or RESOURCE	SOURCE	IDENTIFIER
*Chemicals*		
Clear-sol II	Nacalai Tesque	Cat# 09136-83
[8-^14^C]-Uric acid (53 mCi/mmol)	American Radiolabeled Chemicals	Cat# ARC0513
Dimethyl Sulfoxide	Nacalai Tesque	Cat# 13445-74; CAS: 67-68-5
Ethanol	FUJIFILM Wako Pure Chemical	057-00451; CAS: 64-17-5
Methanol	FUJIFILM Wako Pure Chemical	137-01823; CAS: 67-56-1
*n*-Hexane	FUJIFILM Wako Pure Chemical	085-00416; CAS: 110-54-3
Ethyl acetate	FUJIFILM Wako Pure Chemical	051-00356; CAS: 141-78-6
*n*-Buthanol	FUJIFILM Wako Pure Chemical	026-03326; CAS: 71-36-3
Polyethelenimine “MAX”	Polysciences	Cat# 24765; CAS: 49553-93-7
2′-Hydroxychalcone	Tokyo Chemical Industry	Cat# H0385; CAS: 1214-47-7; Purity: >98%
3-Hydroxyflavone	Tokyo Chemical Industry	Cat# H0379; CAS: 577-85-5; Purity: ≥98%
4-Hydroxychalcone	Tokyo Chemical Industry	Cat# H0955; CAS: 20426-12-4; Purity: >96%
4′-Hydroxychalcone	Tokyo Chemical Industry	Cat# H0945; CAS: 2657-25-2; Purity: >95%
Aesculetin	FUJIFILM Wako Pure Chemical	Cat# A15393; CAS: 305-01-1; Purity: N/A
Apigenin	FUJIFILM Wako Pure Chemical	Cat# 016-18911; CAS: 520-36-5; Purity: ≥95%
Cardamonin	R&D systems	Cat# 2509/10; CAS: 19309-14-9; Purity: ≥98%
Daidzein	FUJIFILM Wako Pure Chemical	Cat# 043-28071; CAS: 486-66-8; Purity: ≥98%
Dihydromyricetin	EXTRASYNTHESE	Cat# 1351-10 mg; CAS: 27200-12-0; Purity: ≥95%
Fisetin	LKT Labs	Cat# F3473; CAS: 528-48-3; Purity: ≥97%
Galangin	ChromaDex	Cat# ASB-00007030-010; CAS: 548-83-4; Purity: N/A
Genistein	FUJIFILM Wako Pure Chemical	Cat# 073-05531; CAS: 446-72-0; Purity: ≥98%
Gossypetin	ChromaDex	Cat# ASB-00007390-010; CAS: 489-35-0; Purity: N/A
Isoliquiritigenin	Tokyo Chemical Industry	Cat# I0822; CAS: 961-29-5; Purity: ≥97%
Kaempferol	FUJIFILM Wako Pure Chemical	Cat# 110-00451; CAS: 520-18-3; Purity: ≥95%
Luteolin	Cayman Chemical	Cat# 10004161; CAS: 491-70-3; Purity: ≥98%
Morin	Combi-Blocks	Cat# QC-0527; CAS: 480-16-0; Purity: ≥98%
Myricetin	FUJIFILM Wako Pure Chemical	Cat# 137-16791; CAS: 529-44-2; Purity: ≥98%
Naringenin chalcone	ChromaDex	Cat# ASB-00014207-005; CAS: 73692-50-9; Purity: N/A
Phloretin	FUJIFILM Wako Pure Chemical	Cat# 160-17781; CAS: 60-82-2; Purity: ≥98%
Quercetagetin	ChromaDex	Cat# ASB-00017020-005; CAS: 90-18-6; Purity: N/A
Quercetin	ChromaDex	Cat# ASB-00017030-010; CAS: 117-39-5: Purity: ≥97%
Taxifolin	EXTRASYNTHESE	Cat# 1036; CAS: 17654-26-1; Purity: N/A
Xanthohumol	TOKIWA PHYTOCHEMICAL	Cat# P2217; CAS: 569-83-5; Purity: ≥98%
***Critical Commercial Assays***		
Pierce BCA Protein Assay Reagent A, B	Thermo Fisher Scientific	Cat# 23223, Cat# 23224
PureLink HiPure Plasmid Filter Midiprep Kit	Thermo Fisher Scientific	Cat# K210015
***Recombinant DNA***		
The complete human URAT1 cDNA in pEGFP-C1	Saito et al. 2020 [14]	NCBI Reference Sequence: NM_144585.3
***Experimental Models: Cell Lines***		
293A	Invitrogen	R70507

N/A, not available.

## Data Availability

Data supporting the findings of this study are included in this published article or are available from the corresponding author on reasonable request.

## Appendix A

**Table A1:** Tested plant materials.

Descriptions in This Study	Common Names	Academic Names	Details of Material *
*Abelmoschus esculentus* (beniokura)	Beniokura	*Abelmoschus esculentus*	Fresh
*Agaricus bisporus*	Common mushroom	*Agaricus bisporus*	Fresh
*Allium cepa*	Onion	*Allium cepa*	Fresh
*Allium oschaninii*	Shallot	*Allium oschaninii*	Fresh
*Allium sativum*	Garlic	*Allium sativum*	Fresh
*Allium sativum* (sprout)	Garlic shoots	*Allium sativum*	Fresh sprout
*Allium tuberosum*	Chinese chive	*Allium tuberosum*	Fresh
*Ananas comosus* (coat)	Pineapple	*Ananas comosus*	Fresh coat
*Apium graveolens*	Celery	*Apium graveolens*	Fresh
*Apium graveolens* (salad celery)	Salad celery	*Apium graveolens*	Fresh
*Arachis hypogaea* (beans)	Peanut	*Arachis hypogaea*	Fresh beans
*Arachis hypogaea* (shell)	Peanut	*Arachis hypogaea*	Fresh shell
*Aralia cordata*	Udo	*Aralia cordata*	Fresh
*Aralia elata* (sprout)	Fatsia sprouts	*Aralia elata*	Fresh sprout
*Arctium lappa*	Edible burdock	*Arctium lappa*	Fresh root
*Arctium lappa* (burdock tea)	Burdock root tea	*Arctium lappa*	Dried root for tea
*Aspalathus linearis*	Rooibos tea leaves	*Aspalathus linearis*	Dried leaves for tea
*Asparagus officinalis* (grass roots)	Asparagus	*Asparagus officinalis*	Fresh grass roots
*Asparagus* spp.	Asparagus	*Asparagus* spp.	Fresh stalk
*Auricularia auricula-judae*	Jew’s ear fungus	*Auricularia auricula-judae*	Fresh
Barley (*Hordeum vulgare*) Miso	Barley Miso	*Hordeum vulgare* ^#^	Japanese traditional fermented product
*Basella alba*	Indian spinach	*Basella alba*	Fresh
*Benincasa hispida* (coat, placenta, seed)	Winter melon	*Benincasa hispida*	Fresh coat, placenta and seeds
*Benincasa hispida* (meat)	Winter melon	*Benincasa hispida*	Fresh meat
*Brassica chinensis*	Green pak choi	*Brassica rapa var. chinensis*	Fresh
*Brassica oleracea* (broccoli, anthotaxy)	Broccoli	*Brassica oleracea var. italica*	Fresh anthotaxy
*Brassica oleracea* (broccoli, sprout)	Broccoli	*Brassica oleracea var. italica*	Fresh sprout
*Brassica oleracea* (broccoli, stem)	Broccoli	*Brassica oleracea var. italica*	Fresh stem
*Brassica oleracea* (kohlrabi, peel)	German turnip or turnip cabbage	*Brassica oleracea var. gongylodes*	Fresh peel
*Brassica oleracea* (red cabbage, sprout)	Red cabbage	*Brassica oleracea var. capitata F. rubra*	Fresh sprout
*Brassica oleracea* (romanesco broccoli, stem)	Romanesco broccoli	*Brassica oleracea var. botrytis*	Fresh stem
*Brassica oleracea* (soft kale)	Soft kale	*Brassica oleracea var. acephala*	Fresh stems and leaves
*Brassica oleracea* (stick senor)	Stick senor	*Brassica oleracea var. italica*	Fresh
*Brassica oleracea* (wild cabbage, flower)	Romanesco broccoli	*Brassica oleracea var. botrytis*	Fresh flower
*Brassica rapa* (ayameyuki-kabu)	Ayameyuki-kabu	*Brassica rapa*	Fresh leaves
*Brassica rapa* (ayameyuki-kabu, meat)	Ayameyuki-kabu	*Brassica rapa*	Fresh meat
*Brassica rapa* (nabana)	Chinese colza	*Brassica rapa* L. *var. nippo-oleifera*	Fresh leaves
*Brassica rapa* (nabana, flower)	Chinese colza	*Brassica rapa* L. *var. nippo-oleifera*	Fresh flower
*Brassica rapa* (red)	Red potherb mustard	*Brassica rapa var. laciniifolia*	Fresh
*Brassica rapa* (santo-sai)	Santo-sai	*Brassica rapa* L. *var. pekinensis*	Fresh
*Capsicum annuum* (redpepper)	Chili pepper	*Capsicum annuum* L.	Fresh
*Capsicum annuum* (sweet pepper)	Sweet pepper	*Capsicum annuum* L. *‘grossum’*	Fresh
*Capsicum annuum* (red)	Red bell pepper	*Capsicum annuum* L. *‘grossum’*	Fresh
*Capsicum annuum* (shishitou)	Shishitou	*Capsicum annuum* L.	Fresh
*Capsicum annuum* (yellow)	Yellow bell pepper	*Capsicum annuum* L. *‘grossum’*	Fresh
*Capsicum frutescens*	Shima pepper	*Capsicum frutescens* L.	Fresh
*Carica papaya* (immature, meat)	Green papaya	*Carica papaya* L.	Fresh meat
*Carica papaya* (immature, peel, placenta, seed)	Green papaya	*Carica papaya* L.	Fresh peel, placenta and seed
*Caulerpa lentillifera*	Sea grape	*Caulerpa lentillifera*	Fresh
*Citrus aurantiifolia* (peel)	Lime	*Citrus aurantiifolia*	Peel
*Citrus depressa* (peel)	Shikuwasa	*Citrus depressa*	Peel
*Citrus junos* (peel)	Yuzu	*Citrus junos*	Peel
*Citrus maxima* (peel)	Pomelo	*Citrus maxima*	Peel
*Citrus maxima* (placenta)	Pomelo	*Citrus maxima*	Inner white and soft tissue layer
*Citrus natsudaidai* (peel)	Suruga elegant	*Citrus natsudaidai*	Peel
*Citrus paradisi* (peel)	Grapefruit	*Citrus paradisi*	Peel
*Citrus reticulata* (peel)	Ponkan	*Citrus reticulata*	Peel
*Citrus sinensis* (blood orange, peel)	Blood orange	*Citrus sinensis*	Peel
*Citrus sinensis* (navel)	Navel	*Citrus sinensis*	Peel
*Citrus sphaerocarpa* (peel)	Kabosu	*Citrus sphaerocarpa*	Peel
*Citrus sudachi* (peel)	Sudachi	*Citrus sudachi*	Peel
*Citrus tangelo* (peel)	Mineola orange (tangelo)	*Citrus tangelo*	Peel
*Cocos nucifera* (young)	Young coconut	*Cocos nucifera*	Fresh
*Colocasia esculenta*	Eddoe	*Colocasia esculenta* L. *schott*	Fresh
*Coriandrum sativum*	Coriander	*Coriandrum sativum*	Fresh leaves
*Coriandrum sativum* (leaves)	Coriander	*Coriandrum sativum*	Fresh leaves
*Cucumis melo* (coat)	Melon	*Cucumis melo*	Fresh coat
*Cucurbita* (meat)	Squash	*Cucurbita*	Fresh meat, without seeds
*Cucurbita* (peel)	Squash	*Cucurbita*	Fresh peel
*Cucurbita pepo* (yellow, peel)	Zucchini	*Cucurbita pepo*	Fresh peel
*Curcuma longa*	Turmeric	*Curcuma longa* L.	Dried powder
*Cyperus esculentus* (powder)	Yellow nutsedge	*Cyperus esculentus*	Milled powder of stem
*Daucus carota*	Carrot	*Daucus carota* subsp. *sativus*	Fresh
*Daucus carota* (purple carrot)	Purple carrot	*Daucus carota* subsp. *sativus*	Fresh
*Dioscorea japonica*	Japanese yam	*Dioscorea japonica*	Fresh
*Diospyros kaki* (shibugaki, meat)	Kaki persimmon	*Diospyros kaki*	Fresh
*Diospyros kaki* (shibugaki, peel)	Kaki persimmon	*Diospyros kaki*	Fresh
*Eriobotrya japonica* (peel)	Loquat	*Eriobotrya japonica*	Fresh
*Eutrema japonicum*	Japanese horseradish	*Eutrema japonicum*	Fresh root
*Eutrema japonicum* (stem)	Japanese horseradish	*Eutrema japonicum*	Fresh stem
*Fagopyrum tataricum*	Tartary buckwheat	*Fagopyrum tataricum*	Dried seed
*Ficus carica*	Fig tree	*Ficus carica*	Fresh fruit
*Flammulina velutipes*	Enoki mushroom	*Flammulina velutipes*	Fresh
*Fortunella* (peel)	Kumquat	*Fortunella*	Peel
*Fragaria ananassa*	Strawberry	*Fragaria ananassa*	Fresh
*Ginkgo biloba* (seed)	Ginkgo	*Ginkgo biloba*	Fresh
*Glebionis coronaria*	Crown daisy	*Glebionis coronaria*	Fresh
*Glycine max*	Soybeans (yellow soybean)	*Glycine max*	Dried product
*Glycine max* (hidenmame)	Soybeans (green soybean)	*Glycine max*	Dried product
*Glycine max* (immature)	Immature soybeans	*Glycine max*	Fresh
*Glycine max* (immature, shuck)	Immature soybeans	*Glycine max*	Fresh shuck
*Glycine max × Bacillus subtilis*	Natto	*Glycine max*	Commercially available Japanese traditional fermented product
*Grifola frondosa*	Hen-of-the-woods	*Grifola frondosa*	Fresh
*Hibiscus rosa-sinensis*	Chinese hibiscus	*Hibiscus rosa-sinensis*	Fresh
*Hosta sieboldiana*	Hosta	*Hosta sieboldiana*	Fresh young leaves
*Houttuynia cordata*	Fish mint	*Houttuynia cordata*	Dried leaves and stem
*Humulus lupulus* (cone)	Hop	*Humulus lupulus*	Frozen hop cone
*Hylocereus undatus* (peel)	Dragon fruit	*Hylocereus undatus*	Fresh peel
*Hypsizygus marmoreus*	Shimeji mushroom	*Hypsizygus marmoreus*	Fresh
*Ilex paraguariensis* (roasted)	Yerba mate tea leaves	*Ilex paraguariensis*	Dried and roasted leaves for tea
*Illicium verum*	Star anise	*Illicium verum*	Dried fruit
*Ipomoea aquatica*	Water morning glory	*Ipomoea aquatica*	Fresh
*Jasminum sambac*	Jasmine tea leaves	*Jasminum sambac*	Dried leaves for tea
*Lactuca sativa*	Stem lettuce	*Lactuca sativa* L. *var. crispa*	Fresh
*Laminaria longissima* (tororomekonbu)	Tororomekonbu	*Laminaria longissima*	Dried product
*Laurus nobilis* (leaves)	Laurel	*Laurus nobilis*	Fresh leaves
*Lentinula edodes*	Shiitake mushroom	*Lentinula edodes*	Fresh
*Lycopersicum esculentum* (yellow)	Cherry tomato	*Solanum lycopersicum* L.	Fresh
*Matricaria recutita*	Chamomile	*Matricaria recutita*	Dried herb product
*Matteuccia struthiopteris* (young)	Ostrich fern	*Matteuccia struthiopteris*	Fresh young leaves
*Mesembryanthemum crystallinum*	Common ice plant	*Mesembryanthemum crystallinum*	Fresh
*Momordica charantia* (coat)	Bitter melon	*Momordica charantia*	Fresh coat
*Musa* spp. (peel)	Banana	*Musa* spp.	Fresh peel
*Musa* spp. (peel, Ecuador)	Banana	*Musa* spp.	Fresh peel
*Nasturtium officinale*	Watercress	*Nasturtium officinale*	Fresh
*Nelumbo nucifera*	Lotus root	*Nelumbo nucifera*	Fresh root
*Ocimum basilicum*	Basil	*Ocimum basilicum*	Fresh
*Ocimum basilicum* (purple)	Purple basil	*Ocimum basilicum*	Fresh
*Oryza sativa* (black)	Brack rice	*Oryza sativa*	Fresh
*Perilla frutescens*	Perilla	*Perilla frutescens*	Fresh
*Persea americana* (coat)	Avocado	*Persea americana*	Fresh coat
*Persea americana* (seed)	Avocado	*Persea americana*	Fresh seed
*Petasites japonicus*	Giant butterbur	*Petasites japonicus*	Fresh
*Petroselinum crispum* (leaves)	Parsley	*Petroselinum crispum*	Fresh leaves
*Phaseolus vulgaris*	Common bean	*Phaseolus vulgaris*	Fresh
*Phaseolus vulgaris* (Moroccan kidney beans)	Moroccan kidney beans	*Phaseolus vulgaris*	Fresh
*Pholiota microspora*	Butterscotch mushroom	*Pholiota microspora*	Fresh
*Phyllostachys pubescens* (young, dried)	Bamboo shoot	*Phyllostachys pubescens*	Dried young stem
*Pisum sativum*	Pea	*Pisum sativum*	Fresh
*Pisum sativum* (shelled)	Shelled pea	*Pisum sativum*	Fresh
*Pisum sativum* (shuck-edible)	Shuck-edible pea	*Pisum sativum*	Fresh
*Pisum sativum* (young leaves)	Pea young leaves	*Pisum sativum*	Fresh
*Pleurotus cornucopiae*	Golden oyster mushroom	*Pleurotus cornucopiae var. citrinopileatus*	Fresh
*Pleurotus eryngii*	King trumpet mushroom	*Pleurotus eryngii*	Fresh
*Pleurotus ostreatus*	Oyster mushroom	*Pleurotus ostreatus*	Fresh
*Prunus domestica* (extract)	Prune extract	*Prunus domestica*	Product of prune pulp extract ^‡^
*Prunus domestica* (meat)	Prune	*Prunus domestica*	Product of prune pulp without seed
*Prunus tomentosa* (peel)	Cherry	*Prunus tomentosa*	Fresh peel
*Psidium guajava* (Chinese)	Guava tea leaves	*Psidium guajava*	Dried leaves for tea cultivated in China
*Psidium guajava* (Japanese)	Guava tea leaves	*Psidium guajava*	Dried leaves for tea cultivated in Japan
*Psophocarpus tetragonolobus*	Winged bean	*Psophocarpus tetragonolobus*	Fresh
*Pteridium aquilinum*	Western bracken fern	*Pteridium aquilinum*	Fresh
*Pyrus communis* (peel)	Pear	*Pyrus communis*	Fresh peel
*Raphanus sativus* (leaves)	Radish	*Raphanus sativus* L. *var. sativus*	Fresh leaves
*Raphanus sativus* (meat)	Radish	*Raphanus sativus* L. *var. sativus*	Fresh meat
*Raphanus sativus* (radish sprout)	Radish sprout	*Raphanus sativus*	Fresh
Rice (*Oryza sativa*) Miso	Rice Miso	*Oryza sativa ^#^*	Japanese traditional fermented product
*Rosmarinus officinalis* (raw)	Rosemary	*Rosmarinus officinalis*	Fresh
*Sechium edule* (meat)	Chayote	*Sechium edule*	Fresh meat
*Sechium edule* (peel, placenta)	Chayote	*Sechium edule*	Fresh peel and placenta
*Sesamum indicum*	Sesame	*Sesamum indicum*	Dried seeds
Siranuhi, (*Citrus unshiu × C. sinensis*) *× C. reticulata* (peel)	Siranuhi	(*Citrus unshiu × C. sinensis*) *× C. reticulata*	Fresh peel
*Smallanthus sonchifolius*	Yacón tea	*Smallanthus sonchifolius*	Dried tea powder
*Smallanthus sonchifolius* (meat)	Yacón	*Smallanthus sonchifolius*	Fresh meat
*Smallanthus sonchifolius* (peel)	Yacón	*Smallanthus sonchifolius*	Fresh peel
*Solanum melongena* (peel)	Aubergine	*Solanum melongena*	Fresh peel
*Vitis labruscana* (peel)	Delaware grapes	*Vitis labruscana*	Fresh peel
*Zanthoxylum bungeanum*	Sichuan pepper	*Zanthoxylum bungeanum*	Dried powder
*Zea mays* (baby corn)	Baby corn	*Zea mays*	Fresh
*Zea mays* (kiritani)	Kiritani	*Zea mays*	Fresh
*Zingiber mioga*	Myoga	*Zingiber mioga*	Fresh
*Zingiber officinale*	Ginger	*Zingiber officinale*	Fresh

*, Unless otherwise indicated, fresh material was used. #, Academic name of main material of Miso product. ^‡^, After defatting via liquid–liquid partition with an equal volume of ethyl acetate, the obtained water phase of extract was subjected to lyophilization.

**Table A2:** Screening of the inhibitory effects of tested plant extracts (20 ppm) on URAT1 function.

Descriptions in This Study	% *	Descriptions in This Study	% *	Descriptions in This Study	% *
*Abelmoschus esculentus* (beniokura)	57.3	*Citrus natsudaidai* (peel)	74.5	*Matricaria recutita*	126.1
*Agaricus bisporus*	38.1	*Citrus paradisi* (peel)	92.4	*Matteuccia struthiopteris* (young)	116.6
*Allium cepa*	32.9	*Citrus reticulata* (peel)	50.2	*Mesembryanthemum crystallinum*	97.4
*Allium oschaninii*	59.5	*Citrus sinensis* (blood orange, peel)	65.2	*Momordica charantia* (coat)	97.8
*Allium sativum*	92.1	*Citrus sinensis* (navel)	81.4	*Musa* spp. (peel)	62.2
*Allium sativum* (sprout)	117.9	*Citrus sphaerocarpa* (peel)	119.1	*Musa* spp. (peel, Ecuador)	94.8
*Allium tuberosum*	77.7	*Citrus sudachi* (peel)	59.3	*Nasturtium officinale*	107.7
*Ananas comosus* (coat)	108.1	*Citrus tangelo* (peel)	34.7	*Nelumbo nucifera*	41.4
*Apium graveolens*	68.7	*Cocos nucifera* (young)	100.2	*Ocimum basilicum*	100.0
*Apium graveolens* (salad celery)	35.8	*Colocasia esculenta*	58.5	*Ocimum basilicum* (purple)	79.4
*Arachis hypogaea* (beans)	38.0	*Coriandrum sativum*	130.2	*Oryza sativa* (black)	67.8
*Arachis hypogaea* (shell)	24.3	*Coriandrum sativum* (leaves)	20.8	*Perilla frutescens*	81.6
*Aralia cordata*	43.9	*Cucumis melo* (coat)	67.3	*Persea americana* (coat)	106.7
*Aralia elata* (sprout)	86.4	*Cucurbita* (meat)	91.5	*Persea americana* (seed)	63.8
*Arctium lappa*	59.9	*Cucurbita* (peel)	66.5	*Petasites japonicus*	64.3
*Arctium lappa* (burdock tea)	97.4	*Cucurbita pepo* (yellow, peel)	37.8	*Petroselinum crispum* (leaves)	75.7
*Aspalathus linearis*	29.0	*Curcuma longa*	83.7	*Phaseolus vulgaris*	132.6
*Asparagus officinalis* (grass roots)	48.1	*Cyperus esculentus* (powder)	99.2	*Phaseolus vulgaris* (Moroccan kidney beans)	140.3
*Asparagus* spp.	77.0	*Daucus carota*	55.1	*Pholiota microspora*	55.4
*Auricularia auricula-judae*	31.4	*Daucus carota* (purple carrot)	52.0	*Phyllostachys pubescens* (young, dried)	47.4
Barley (*Hordeum vulgare*) Miso	105.5	*Dioscorea japonica*	44.8	*Pisum sativum*	62.2
*Basella alba*	63.5	*Diospyros kaki* (shibugaki, meat)	112.0	*Pisum sativum* (shelled)	107.8
*Benincasa hispida* (coat, placenta, seed)	50.3	*Diospyros kaki* (shibugaki, peel)	51.7	*Pisum sativum* (shuck-edible)	50.2
*Benincasa hispida* (meat)	25.2	*Eriobotrya japonica* (peel)	109.7	*Pisum sativum* (young leaves)	49.3
*Brassica chinensis*	93.8	*Eutrema japonicum*	73.7	*Pleurotus cornucopiae*	90.7
*Brassica oleracea* (broccoli, anthotaxy)	22.7	*Eutrema japonicum* (stem)	102.2	*Pleurotus eryngii*	49.9
*Brassica oleracea* (broccoli, sprout)	61.4	*Fagopyrum tataricum*	36.5	*Pleurotus ostreatus*	73.1
*Brassica oleracea* (broccoli, stem)	68.5	*Ficus carica*	79.1	*Prunus domestica* (extract)	84.4
*Brassica oleracea* (kohlrabi, peel)	73.4	*Flammulina velutipes*	39.7	*Prunus domestica* (meat)	96.3
*Brassica oleracea* (red cabbage, sprout)	106.2	*Fortunella* (peel)	62.5	*Prunus tomentosa* (peel)	49.5
*Brassica oleracea* (romanesco broccoli, stem)	109.8	*Fragaria ananassa*	52.0	*Psidium guajava* (Chinese)	73.4
*Brassica oleracea* (soft kale)	94.4	*Ginkgo biloba* (seed)	40.2	*Psidium guajava* (Japanese)	52.5
*Brassica oleracea* (stick senor)	51.2	*Glebionis coronaria*	17.9	*Psophocarpus tetragonolobus*	90.7
*Brassica oleracea* (wild cabbage, flower)	60.0	*Glycine max*	66.4	*Pteridium aquilinum*	192.2
*Brassica rapa* (ayameyuki-kabu)	108.2	*Glycine max* (hidenmame)	104.0	*Pyrus communis* (peel)	58.8
*Brassica rapa* (ayameyuki-kabu, meat)	85.2	*Glycine max* (immature)	64.6	*Raphanus sativus* (leaves)	70.8
*Brassica rapa* (nabana)	86.1	*Glycine max* (immature, shuck)	96.1	*Raphanus sativus* (meat)	64.7
*Brassica rapa* (nabana, flower)	64.2	*Glycine max × Bacillus subtilis*	60.6	*Raphanus sativus* (radish sprout)	79.6
*Brassica rapa* (red)	52.6	*Grifola frondosa*	69.5	Rice (*Oryza sativa*) Miso	58.6
*Brassica rapa* (santo-sai)	72.2	*Hibiscus rosa-sinensis*	97.5	*Rosmarinus officinalis* (raw)	13.6
*Capsicum annuum* (redpepper)	83.0	*Hosta sieboldiana*	45.2	*Sechium edule* (meat)	130.0
*Capsicum annuum* (sweet pepper)	107.8	*Houttuynia cordata*	84.0	*Sechium edule* (peel, placenta)	52.0
*Capsicum annuum* (red)	80.3	*Humulus lupulus* (cone)	78.9	*Sesamum indicum*	158.6
*Capsicum annuum* (shishitou)	73.0	*Hylocereus undatus* (peel)	120.0	Siranuhi, (*Citrus unshiu × C. sinensis*) *× C. reticulata* (peel)	52.7
*Capsicum annuum* (yellow)	81.4	*Hypsizygus marmoreus*	58.1	*Smallanthus sonchifolius*	35.4
*Capsicum frutescens*	58.8	*Ilex paraguariensis* (roasted)	69.1	*Smallanthus sonchifolius* (meat)	74.3
*Carica papaya* (immature, meat)	78.5	*Illicium verum*	24.6	*Smallanthus sonchifolius* (peel)	110.5
*Carica papaya* (immature, peel, placenta, seed)	94.3	*Ipomoea aquatica*	75.5	*Solanum melongena* (peel)	133.8
*Caulerpa lentillifera*	65.5	*Jasminum sambac*	66.5	*Vitis labruscana* (peel)	150.9
*Citrus aurantiifolia* (peel)	185.5	*Lactuca sativa*	91.6	*Zanthoxylum bungeanum*	57.2
*Citrus depressa* (peel)	38.9	*Laminaria longissima* (tororomekonbu)	112.2	*Zea mays* (baby corn)	65.3
*Citrus junos* (peel)	61.6	*Laurus nobilis* (leaves)	33.6	*Zea mays* (kiritani)	78.1
*Citrus maxima* (peel)	64.7	*Lentinula edodes*	62.8	*Zingiber mioga*	28.0
*Citrus maxima* (placenta)	68.9	*Lycopersicum esculentum* (yellow)	60.6	*Zingiber officinale*	57.3

*, Data for URAT1-mediated urate transport are expressed as % of the vehicle control (1% dimethyl sulfoxide) (*n* = 1, each sample). Results for the top 40 samples are shown in Figure 1.
